# Improving Mental Health Help-Seeking Behaviours for Male Students: A Framework for Developing a Complex Intervention

**DOI:** 10.3390/ijerph17144965

**Published:** 2020-07-09

**Authors:** Ilyas Sagar-Ouriaghli, Emma Godfrey, Selina Graham, June S. L. Brown

**Affiliations:** 1 Department of Psychology, Institute of Psychiatry, Psychology and Neuroscience (IoPPN), King’s College London, London SE5 8AF, UK; emma.l.godfrey@kcl.ac.uk; 2 Department of Population Health and Environmental Sciences, Faculty of Life Sciences and Medicine, King’s College London, London SE1 9RT, UK; 3 School of Cancer and Pharmaceutical Sciences, King’s College London, London SE1 9NH, UK; selina.graham@kcl.ac.uk

**Keywords:** help-seeking, men, interventions, students, mental health, COM-B, MRC complex intervention

## Abstract

Men are less likely to seek help for mental health difficulties and this process is often used to help explain the disproportionally higher suicide rates compared to women. Furthermore, university students are often regarded as a vulnerable population group with a lower propensity to seek help. Thus, male students are a very high-risk group that is even more reluctant to seek help for mental health difficulties, placing them at high risk of suicide. Often, student mental health problems are highlighted in the media, but very few evidence-based solutions specifically designed for male students exist. The current paper seeks to provide a comprehensive framework about how to better design mental health interventions that seek to improve male students’ willingness to access psychological support. The Medical Research Council’s (MRC’s) framework for developing a complex intervention was used to develop an intervention relevant to male students. In this paper, previous help-seeking interventions and their evaluation methods are first described, secondly, a theoretical framework outlining the important factors male students face when accessing support, and thirdly, how these factors can be mapped onto a model of behaviour change to inform the development of an evidence-based intervention are discussed. Finally, an example intervention with specific functions and behaviour change techniques is provided to demonstrate how this framework can be implemented and evaluated. It is hoped that this framework can be used to help reduce the disparity between male and female students seeking mental health support.

## 1. Introduction

In 2018, 33% of 18-year-olds enrolled into university education in the United Kingdom (UK) [1]. This period coincides with the peak onset age for various mental health conditions, such as schizophrenia, anxiety and depression [2,3]. Anxiety and depression occur frequently in university students and are often caused or exacerbated by concerns relating to academic performance, pressure to succeed and post-graduation plans [4]. This places students at a greater risk of experiencing psychological difficulties with suicidal thoughts and behaviours reported in just under a quarter (22%) of this population group [5]. Although female students are more likely to be diagnosed with depression and anxiety and frequently report suicidal thoughts/behaviours [5,6,7], 69% of suicides in 2015 were completed by male students [8]. For younger men aged between 15–29 years old, suicide is the second leading cause of death [9,10].

Explanations for this phenomenon are often associated with willingness to seek help for mental health difficulties. Young people aged 16–24 overall represent the least likely age group to receive mental health treatment [11]. Additionally, male students are less likely to seek help compared to female students [12]. Female students are significantly more likely to use mental health services than male students (OR = 1.54) [13]. This trend continues into later adulthood, whereby only 9% of men receive treatment for a mental health condition compared to 15% of women [11], with women remaining 1.58 times (95% CI 1.32 to 1.89) more likely to receive mental health treatment compared to men even after controlling for prevalence rates [14]. This helps explain why globally adult men are 2.35 more likely to take their own life compared to women [9], or even up to 3.5 times more likely in high-income countries such as the UK [9,10]. Therefore, reducing help-seeking barriers for male students and engaging them with mental health initiatives can not only improve health outcomes whilst at university but can also have a preventative function and lead to more positive help-seeking behaviours in adulthood.

Although both male and female students face a range of barriers to seeking psychological support [15,16], lower rates of help-seeking observed in men are often attributed to traditional stereotypes of masculinity including, stoicism, self-reliance, and restrictive emotionality [17,18]. For instance, male students may view seeking support and expressing one’s emotions as a sign of weakness, whilst it is deemed acceptable for female students to express and articulate themselves emotionally [19]. Moreover, male students prefer to limit emotional disclosure and deny weakness as a way to preserve their autonomy and stoicism [20]. Due to poor help-seeking in male university students and their increased risk of suicide, universities are faced with increasing pressure to implement mental health initiatives, which may mean they are not necessarily evidence based or gender appropriate [21]. Only a handful of evidence-based interventions targeting help-seeking for men have been evaluated [22], with even fewer targeted specifically towards male university students [23,24]. When such strategies are published, the intervention development process is not reported. It is essential for the intervention development process to be reported as this can enhance our theoretical and practical understanding about developing mental health interventions for male students [25]. In response to this, the current paper seeks to develop the first framework for developing and designing mental health interventions for male students that is grounded in evidence-based practice.

## 2. Medical Research Council (MRC) Framework

**Figure 1:**
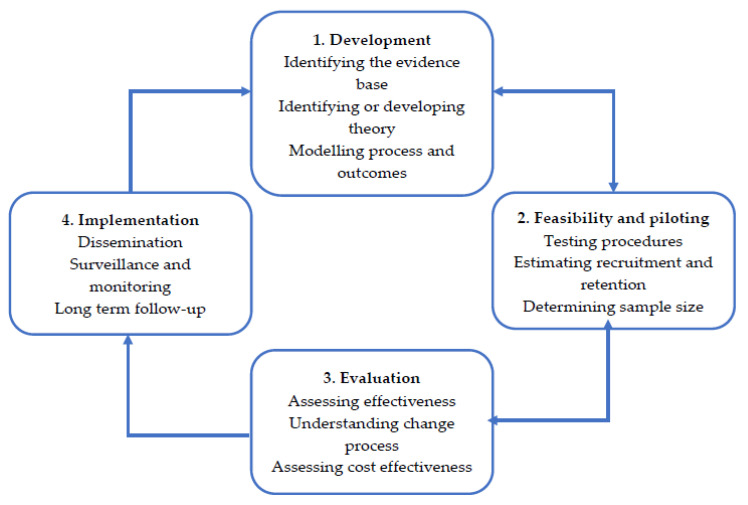
Medical Research Council’s (MRC’s) four key stages for developing and evaluating an intervention [26].

To develop an intervention targeting help-seeking behaviours in male students, the MRC’s framework for developing a complex intervention was adhered to [26,27]. The MRC framework has four key stages, consisting of development, feasibility and piloting, evaluation, and implementation (Figure 1) [26]. As of 2019, this framework was updated, with additional action points being added for the development stage of the original framework [27]. Although these action points need to be considered when developing an intervention, not all of the actions can be addressed nor are relevant to every problem or context [27]. Furthermore, the updated MRC guidelines for developing a complex intervention advises that an approach to intervention development is decided upon first. This paper will discuss the development of an intervention using a published approach grounded in theory and evidence base by combining published research evidence and existing theories [27]. The MRC framework for developing and evaluating complex interventions will be used with the Behaviour Change Wheel to develop a framework for new interventions that addresses help-seeking in male students. It is anticipated that this framework will create a starting point for future interventions, which can be refined as the current evidence base is enriched. Furthermore, specific detail in accordance with the Guidance for Reporting Intervention Development Studies in Health Research (GUIDED) checklist has been included (Appendix A) to further enrich the quality of evidence that is reported within the current paper [25].

### 2.1. MRC Development: Identifying the Evidence Base.

The first stage to consider when developing a complex intervention is to summarise what is already known about similar interventions and the methods that have been used to evaluate them [26,27]. The MRC framework stresses that if a recent systematic review of similar interventions is not available then a high-quality systematic review should be conducted and updated [26]. At the time, there was only one systematic review that summarised six interventions targeting help-seeking for depression, anxiety, and general psychological distress for both males and females across multiple age groups [28]. Of these, three were delivered to university students with a total sample size of 547 (32% male). These interventions included information targeting mental health literacy, content to reduce mental health stigma, service information, and a supportive interview centred around consulting a sports psychologist [29,30,31]. Despite these interventions targeting help-seeking in students, they were not investigating male students specifically. This information is essential for the development of an appropriate intervention for male students as they hold more negative attitudes than female students and are less likely to engage with mental health services [32].

In response to this, a systematic review investigating help-seeking interventions specifically in males was conducted [22]. This systematic review identified nine interventions targeting mental health help-seeking in men of different age groups, two of which were delivered to male students [23,24]. Despite these interventions leading to positive changes in help-seeking, theoretical frameworks leading to their development had not been outlined. This presents difficulties for replication, as well as challenges in identifying what techniques have been key to positive changes [25,33].

As the systematic review conducted by Sagar-Ouriaghli et al. [22] consisted of nine interventions with heterogenous clinical populations and dissimilar designs, a meta-analysis could not be conducted [34]. To provide a coherent summary, a novel method that identified the Behavioural Change Techniques (BCTs) was used [35]. BCTs characterise the smallest identifiable “active ingredients” embedded within an intervention designed to change the desired behaviour [35]. Thus, the key elements that are likely to contribute to improvements in help-seeking behaviours were extracted from these nine interventions [22]. Sagar-Ouriaghli et al. [22] identified 18 BCTs (e.g., credible source, feedback on behaviour and problem solving), which were synthesised into seven broader, more clinically relevant, psychological processes that are likely to contribute to changes in help-seeking for men of different age groups (Appendix B). These seven key processes include: the use of role models (e.g., celebrities and other men) to convey information, psycho-educational materials to improve mental health knowledge, assisting men to recognise and manage their symptoms, adopting active problem solving and/or solution focused tasks, motivating behaviour change, sign-posting mental health services, and finally, including content to build on positive masculine traits (e.g., responsibility and strength).

The identification of these seven processes captured from the nine interventions included the two interventions that target help-seeking behaviours in male-students [23,24]. Furthermore, the three interventions targeting help-seeking behaviours in both male and female students identified by Gulliver et al. [28] confirm the seven key processes identified through the BCTs (Appendix B). In sum, the previous systematic reviews by Sagar-Ouriaghli et al. [22] and Gulliver et al. [28] have captured key processes or elements within interventions that are likely to improve mental health help-seeking for male students.

#### Identifying Evaluation Methods

In addition to identifying previous interventions, The MRC framework emphasises the importance of identifying the methods that have been used to evaluate them [26,27]. Across the 12 interventions outlined above, ten help-seeking measures were utilised. Of these measures, the Attitudes Towards Seeking Professional Psychological Help scale-short form (ATSPPH-SF) [36], was the most commonly used instrument to measure help-seeking, which was used to evaluate four interventions [30,37,38,39].

The initial ATSPPH-long form (ATSPPH-LF) has been validated in 960 students (49% male) demonstrating good internal consistency (α = 0.86) and test–retest reliability (0.82) [40]. The ATSPPH-SF contains ten items taken from the ATSPPH-LF and has demonstrated moderate internal consistency (α = 0.77–0.84), good test-retest reliability (0.80) for university students, and correlates well with the original scale (*r* = 0.87) [36,41]. Higher ATSPPH-SF scores (i.e., more favourable attitudes to help-seeking) and recent mental healthcare use share a significant positive relationship, suggesting that the scale may predict whether someone will access future treatment [41]. Overall, the ATSPPH-SF is an appropriate scale to measure help-seeking attitudes in a male-student population.

In conjunction with help-seeking attitudes, it is also important to capture changes to behavioural or actual help-seeking, such as presenting to a service or reaching out to someone for support. From previous work identified, three studies measured behavioural help-seeking with a psychometric instrument using the Help-Seeking Behaviour Scale (HSBS) or the General Help-Seeking Questionnaire (GHSQ) [24,39]. Of these, only the GHSQ has been validated, making it the preferred and more psychometrically robust instrument to use [42]. The GHSQ is a 24-item scale that assesses future help-seeking intentions/attitudes as well as recent and past help-seeking experiences [43]. The GHSQ has been validated in 218 students aged 12–19 years old (51% male), whilst demonstrating good internal consistency (α = 0.70–0.85) and test–retest reliability over a three-week period (0.86–0.92) [42]. The last ten items of the GHSQ assess recent help-seeking behaviours in the past 2 weeks and is referred to as the Actual Help-Seeking Questionnaire (AHSQ) [44,45]. Overall, two evaluation methods demonstrating good psychometric properties have been identified. Future mental health help-seeking interventions for male students should seek to measure changes to help-seeking attitudes (ATSPPH-SF) and help-seeking behaviours (AHSQ).

### 2.2. MRC Development: Identifying or Developing Theory

Following the identification of previous interventions and evaluation methods, the MRC framework stresses the importance of identifying or drawing upon theory to help identify what is important, relevant, and feasible for an intervention [26,27]. To achieve this, the “access to care model” [46] shall be discussed in the context of barriers male students face when engaging with mental health services.

#### 2.2.1. Access to Care Model

**Figure 2:**
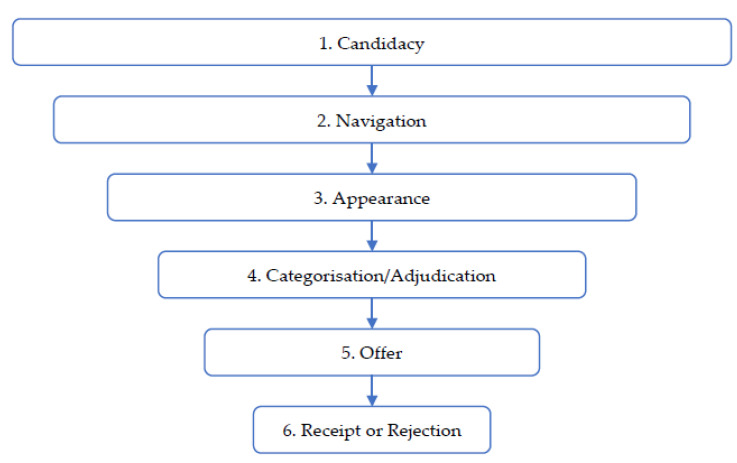
Access to care model.

The access to care model of Gask et al. [46] is a theoretical model outlining how people with common mental health problems (i.e., anxiety and depression) engage with services and is best described within the development stage of the MRC’s framework for developing a complex intervention. The model draws heavily upon an interpretive synthesis of literature summarising healthcare access by vulnerable groups and identifies six key issues with “candidacy” at its core (Figure 2) [47].

##### Candidacy

Candidacy is a dynamic and constantly evolving construct to describe how people’s eligibility for medical intervention is negotiated between themselves and health professionals [47]. Candidacy is focused with one’s role and personal identity, whereby service engagement will occur if it remains congruent with their identity, or that help-seeking will not threaten their competence to fulfil social roles [46].

In the context of male help-seeking, engaging with mental health services can threaten one’s masculinity, impacting both their personal identity and social role(s). Masculine stereotypes are centred around stoicism, emotional control, power, success, and independence [17]. Help-seeking may not align with these stereotypes, as men must control their emotions, be self-sufficient, and endure pain [48]. Seeking help is seen as a loss of control and independence, whilst demonstrating weakness and vulnerability for not being able to cope with emotional distress [48,49]. Indeed, male students who demonstrate higher conformity to masculine norms have greater negative attitudes towards help-seeking [50,51,52,53].

Deviation from these masculine stereotypes can be perceived as non-normative and thus elicit self-stigmatising beliefs or negative perceptions from the wider public [54]. Male students are more likely to report higher public and self-stigma of mental health compared to female students and are thus less likely to use mental health services [55,56]. Although both public- and self-stigma may impact help-seeking, self-stigma is likely to be a stronger predictor than public stigma [55,57,58]. Indeed, self-stigma has been shown to mediate the relationship between conformity to masculine norms and help-seeking amongst male students [52,59,60]. This factor explains why male role models contribute to positive changes in help-seeking as they can assist at an early stage of the help-seeking process with re-aligning mental health help-seeking to be congruent with masculine stereotypes and reduce mental health stigma [22,61,62].

##### Navigation

If help-seeking is not perceived to threaten one’s identity and social role, the individual will then seek to gain entry to a mental health service, referred to as “navigation” [46]. At this stage, the individual needs to rely on their sense of self-efficacy and their mental health literacy to determine their current needs and approach an appropriate service.

Male students may struggle at this stage, particularly for mental health, as they are required to identify services organised around professional psychiatric and psychological models. This can be a particular issue as men have greater difficulty at identifying mental health symptoms compared to women [63,64,65]. Difficulties in identifying mental health symptoms can be explained by poorer mental health literacy, perceiving symptoms as minor or insignificant, or difficulty in associating atypical symptoms with more conventional definitions [49]. Men may be more irritable, violent, and more inclined to engage in substance abuse, which are often regarded as male depressive symptoms [50,66]. Moreover, tolerating a high degree of distress is considered manly and one must only seek help when the problem is serious [48]. Indeed, by definition, conformity to masculine gender roles raises the threshold for when one can express distress, but can also result in denial, undervaluation and failure to identify symptoms that indicate the need for support [67].

Men also experience higher levels of fear and embarrassment associated with the use of services [49]. This arises from the unfamiliarity of healthcare services, the perception of positioning themselves in a vulnerable situation, and being perceived as weak [49]. Sign-posting services sensitively is therefore an important technique to include in future interventions as male students need more information regarding mental health services and who they can contact [22,49].

##### Appearance

The next step of “appearance” requires men being able to identify presenting symptoms through adequate mental health literacy and to identify an appropriate service. Presenting to a service is often left to be the responsibility of the patient, whereby they must initiate contact via their General Practitioner (GP) or self-referring to a relevant mental health service such as Improving Access to Psychological Therapies (IAPT). Another method includes “invitations”, where the patient responds to an invite from a particular service. Similarly, “grabs” remove the component of candidacy by taking away the patient’s control. An example of this includes compulsory mental health screenings done in the workplace or during other physical health appointments.

Despite these avenues, male students may experience fewer opportunities at this stage. Compared to women, men consult medical professionals less often across all age groups [68], with the largest discrepancy occurring in men aged between 21–39 (OR = 0.40). This is often attributed to higher reproductive health appointments seen in women [68]. However, this pattern is still found in men under 21 (i.e., students) (OR = 0.77) and for health check-ups not related to reproductive health (e.g., blood pressure) [68,69]. Consequently, this reduces the opportunity to detect symptoms relating to mental health and facilitate the help-seeking process. To combat this, male friendly services, extended opening hours, and mental health workplace/university programmes may assist with encouraging male students to present to services or provide an increased opportunity for “invitations” and “grabs” [70,71].

##### Categorisation/Adjudication and Offer

Categorisation/adjudication is the next stage whereby a professional judgement is made that either confirms the patient’s illness or confirms their suitability to be offered an appropriate intervention.

Male students may present with atypical symptoms and have difficulties with understanding how these relate to psychological models of poor mental health. This may obscure detection from mental health professionals and diagnostic measures. Moreover, certain symptoms such as aggression and substance abuse may prevent confirmation of distress and brand male students as unsuitable for treatment [72,73]. Additionally, clinicians may hold their own gender biases further inhibiting male students from receiving an offer for mental health treatment [74]. Biases may include, perceiving men as feminine for expressing themselves [75], overlooking men’s emotions, and shaming them for expressing vulnerability by over-stressing independence [74].

These factors all reduce the chances of male students receiving an offer of help and exacerbate the gender differences seen in mental health help-seeking. However, if they are deemed appropriate for treatment an offer will be made, moving them into the final stage of the access to care model.

##### Receipt or Rejection

Receiving an offer for treatment does not guarantee the student will engage as the offer may be rejected. This can be a significant obstacle for men. Only 36% of referrals made to IAPT in 2018 were male, with 36% of 18–35-year old’s declining the referral and disengaging from treatment [76]. For all ages below 65 years, men were less likely to enter and complete treatment compared to women [76].

Furthermore, there is evidence highlighting differences in treatment preferences for both men and women [77]. Women tend to prefer psychotherapy and counselling more than men, whereas men have a greater preference for support groups and occupational support [77,78]. Similarly, men demonstrate higher levels of engagement towards gender-sensitive and proactive (i.e., solution focused) therapies [78,79].

As men have a tendency to delay help-seeking until the severity of symptoms become unmanageable [48,80], a stepped care approach that delivers the least intensive treatment first may be ineffective for men [81,82]. Thus, men with severe symptoms may be offered treatment that is not intensive enough for their current symptoms. These factors all have a part to play in the decision male students make when accepting or rejecting a mental health service/treatment offer.

#### 2.2.2. Other Considerations

Alongside the access to care model, other factors may also be important. Aspects of the male archetype can be positive when facing emotional adversity [83]. Ideals of regaining control via information and relying on one’s owns resources can be helpful strategies for men with mental health difficulties [84]. Englar-Carlson and Kiselica’s [85] positive psychology/positive masculinity model (PPPM) highlights the strengths associated with masculine stereotypes and that men do and will engage with services if male specific issues and approaches are considered [85,86]. Positive masculinity could therefore be used to develop more male student-friendly services [78].

Some strategies for improving engagement have been reviewed [87] and recommendations made by clinicians. These include, clarifying treatment structure, adopting goal-focused or action-oriented approaches, forming collaborative relationships and tailoring language accordingly [87]. Outlining the treatment structure can help to overcome men’s ambivalence, fear, or embarrassment towards help-seeking whilst mitigating client mistrust, suspicion, or fear of dependency within the therapeutic relationship [87]. Clinicians who self-disclose, use person-centred approaches, and focus on strengths can also reduce the client–clinician gap. This assists with building strong therapeutic alliances that are more collaborative, allowing for greater trust and honesty later on. Furthermore, goal-focused or action-oriented approaches can help maintain men’s motivation and engagement with treatment [87]. Similarly, using lay language such as swearing and the appropriate use of humour can assist with forming a collaborative and equal therapeutic relationship [78,88].

Finally, when examining help-seeking facilitators within a student population, positive past experiences, social support or positive encouragement from others, confidentiality and trust in services, positive relationships with services, good mental health literacy, perceiving the problem as serious, and emotional competence have been identified as key factors that encourage students to seek psychological support [15,43,89,90].

### 2.3. MRC Development: Modelling Process and Outcomes

The third step in the development stage of developing a complex intervention in accordance with the MRC’s framework is modelling process and outcomes [26]. Modelling seeks to conduct preliminary testing of an intervention to understand the context in which the intervention will operate and be implemented [27,91]. As a result, a more practical and appropriate intervention can be designed. To understand the context of a male student mental health help-seeking intervention, a series of focus groups were conducted [92].

#### 2.3.1. Modelling Process and Outcomes: Focus Groups

**Figure 3:**
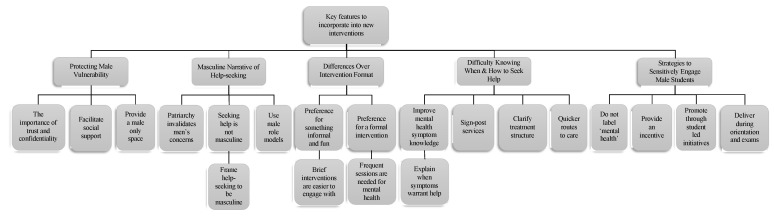
Overview of themes and sub-themes identified from focus groups [92].

The focus groups sought to identify key features of the context that can be incorporated into mental health initiatives to help encourage male students to seek help for mental health difficulties [92]. Three focus groups with 24 male students (mean age of 21.89 years) from a UK London University were asked questions exploring the barriers to seeking help, what would encourage help-seeking, how an intervention should be designed, and how to publicise this intervention to male students. The results from the focus group revealed five themes that male students considered important when designing male-friendly interventions that addressed mental health help-seeking [92]. These themes were: (1) protecting male vulnerability, (2) provide a masculine narrative of help-seeking, (3) preferred intervention formats regarding formality and length (where participants differed), (4) difficulty knowing when and how to seek help, and (5) strategies to sensitively engage male students (Figure 3).

These findings support much of the evidence relating to the influence of masculinity on help-seeking, low mental health literacy, and the need for information about services. Additionally, these focus groups captured more nuanced practical findings that have not been mentioned within the wider literature. This included discrepancies over the formality and duration of interventions and appropriate ways of promoting mental health initiatives to male students.

While both the formality and duration of an intervention are important factors to consider when designing interventions, it was clear that male students differed in their preference, with half stating that they would be more likely to engage in a formal intervention. This was due to the serious nature of mental health difficulties and would provide validation of men’s mental health difficulties [92]. Equally, however, others stated that an informal intervention would be more acceptable. This may allow for greater use of lay language and humour when working with male students [78,88]. Similarly, an informal setting is more familiar to men when building relationships which can help create greater rapport and trust [93]. Certainly, it would be worthwhile to compare the differences in uptake between formal and informal interventions.

There was also a lack of consensus regarding the duration of an intervention. Some students preferred a brief and short intervention (e.g., two sessions lasting up to 2 h each), whilst others requested something more frequent and long standing [92]. Traditionally, 6–12 weekly therapy sessions are considered the gold standard when treating depression and anxiety [94] but referral rates for men remain relatively low [95]. Furthermore, not having enough time is often a key barrier for students wanting to access mental health care [96,97]. Considering these points, a brief intervention provides a more feasible and practical solution (i.e., less time needed) to facilitate help-seeking in male students that existing services fail to offer, possibly as a bridge into pre-existing services.

When engaging male students with mental health initiatives, focus group participants advised against using mental health labels and the term “well-being” [92]. This relates to the finding that men often reject the use of psychological support if it seeks to label emotional distress as a psychiatric illness within a diagnostic framework [98]. Avoiding the use of mental health labels when promoting an intervention allows for a wider reach of male students who do not identify as having a mental health issue or who are experiencing difficulties that are not typically associated with psychological distress [50]. This further reinforces the use of lay language when working with male students.

Advertising mental health initiatives through pre-existing student bodies was advised by the focus groups [92]. Indeed, male students are more likely to seek in-person mental health support when encouraged by a family member or partner, whilst peer encouragement has a greater influence after adolescence—coinciding with university enrolment [99].

A third approach that may elicit higher levels of engagement would be to provide a more direct and immediate incentive. Here, male students perceive engaging with mental health support to be a “net-loss” regarding their masculine identity, time, and other priorities (e.g., university work) [92]. By providing an immediate incentive, such as monetary incentive, fun social bonding, or academic support may help tip this cost–benefit analysis more favourably [92]. This facilitates better opportunity for “appearance” and “invitations” to mental health initiatives as discussed earlier within the Access to Care Model [46].

A final nuanced point that male students unanimously agreed upon was that mental health initiatives should be delivered during the start of an academic year (also known as freshers) and during exam periods [92]. At the start of university, students have more time available to engage with mental health initiatives. Similarly, during exam times, mental health support may be perceived as having a more direct benefit due to exam-related stress [92].

This paper has summarised previous systematic reviews of help-seeking interventions, theory that influences help-seeking in male students, and qualitative work exploring intervention development. Data from the previous interventions, qualitative work and clinical recommendations results in 17 factors that are seen to be very important in changing behaviours relating to help-seeking in male students (Table 1). Additionally, five tools which may assist with changing or improving some of these factors have been discussed. These include the use of role models, sign-posting services, better availability of services, positive masculinity, and the use of humour and lay language.

#### 2.3.2. Modelling Process and Outcomes: The COM-B Model of Behaviour

**Figure 4:**
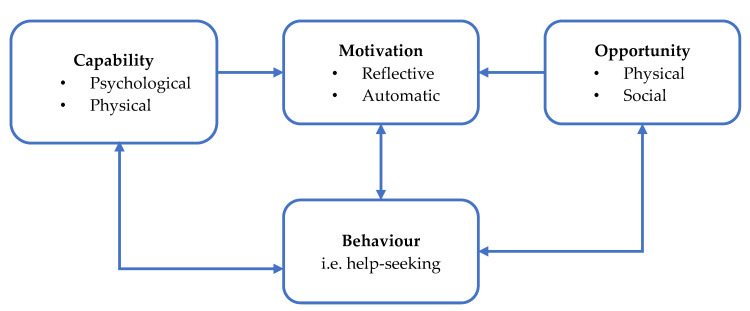
Capability, Opportunity, and Motivation model of Behaviour (COM-B) System.

Following the identification of these factors, it is important that they are implemented and operationalised appropriately. To do so, the Capability, Opportunity, and Motivation model of Behaviour (COM-B) was selected as it has predictive validity on the delivery of behaviour change interventions [100,101]. The COM-B model is a behaviour system that draws on the interaction between capability, opportunity, and motivation to generate a behaviour, in this case help-seeking [100]. Capability refers to the individual’s psychological and physical capacity to engage in the behaviour and is dependent on their knowledge and skills. Motivation encapsulates all brain processes that energise and direct behaviour, further divided into reflective motivation (i.e., conscious evaluation and planning) and automatic motivation (i.e., emotions or impulses that arise from associative learning and/or innate dispositions). Lastly, opportunity includes factors that lie outside the individual that facilitate the behaviour or prompt it, containing both physical and social factors [100] (Figure 4). All of these six domains are strong predictors of the practical delivery of health care professional practice [101].

This behavioural system can then be linked to wider intervention functions and policy categories to help assist with developing appropriate interventions [100,102]. This behaviour change system can be depicted by three layers within the ‘Behaviour Change Wheel’ (BCW) with sources of behaviour (i.e., COM-B domains) at its core, surrounded by intervention functions and lastly policy categories. Similar to the COM-B model, the layers within this system are not linear and each layer component may interact with one another. By using the COM-B model and BCW, it was possible to map the 17 factors that influence help-seeking (Table 1) according to capability, motivation, and opportunity (Table 2). Mapping these factors to their respective domains was completed by two authors (ISO and SG) in an independent parallel fashion before discussing discrepancies to reach 100% consensus. 

Mapping help-seeking factors to the COM-B model provides greater guidance and clarity as to how to improve help-seeking in male students via the intervention function as indicated by the BCW [103]. Firstly, intervention functions that address psychological capability should be focused around education, training, or the enablement of male students to improve their knowledge and awareness of mental health symptoms and services [103]. Secondly, physical opportunity highlights the disparity between male-student needs and the design of pre-existing mental health services. Therefore, intervention functions should include training, restriction, environmental restructuring, and better enablement of mental health services to make them more accommodating for male students [103]. This may include adjusting the availability of services through workplace/academic programmes, extended opening hours [70,71], or by re-structuring therapeutic environments that are shorter and more conducive to building trust and good patient–clinician relationships. Thirdly, reflective motivation appears rooted in male students’ ambivalence toward seeking help. Intervention functions should include education, persuasion, incentivisation, or coercion to elicit more positive evaluations of using psychological support [103]. Finally, social opportunity highlights a wider, more systemic issue regarding notions of masculinity, public stigma and the clinician’s role within therapy. Intervention functions should be rooted in restriction, environmental restricting, modelling, and enablement [103]. Similarly, the training of clinicians may help to reduce clinician bias [74,75]. Furthermore, some of these factors may overlap across multiple domains within the COM-B model. Greater severity of symptoms may increase one’s awareness of their mental state (psychological capability) or may provide a better opportunity for evaluation and planning (reflective motivation). Both the factors of treatment being too time consuming and the preference for proactive therapies can be a perception/evaluation of existing treatments (reflective motivation) or a physical barrier that does not accommodate men’s needs without offering an alternative choice (physical opportunity). Lastly, not all intervention functions should be implemented, and should be chosen based on their affordability, practicability, effectiveness/cost-effectiveness, acceptability, side-effects/safety, and equity—otherwise known as the APEASE criteria [103].

Once an intervention’s functions have been decided upon, the next step requires the identification of the intervention’s content regarding specific techniques that can be operationalised and incorporated into an intervention. This is an iterative process that involves identifying a range of specific techniques from the Behavioural Change Techniques Taxonomy (BCTTv1) [35] that could be considered for any particular function [103]. Once all potential BCT’s have been identified, the APEASE criteria is used once more to determine which specific techniques or tools are most appropriate. Additionally, BCTs that have been frequently used before in similar interventions may also aid in this decision [22,103].

## 3. MRC Feasibility and Piloting

Once all intervention functions, policy categories, and BCTs have been selected, it is possible to then draft an intervention that targets the desired behaviour change, in this case help-seeking. In turn, this enables the newly developed intervention to be evaluated and piloted accordingly. For the purpose of this report, an example intervention is provided that draws upon nine factors that influence male-student help-seeking behaviours for mental health (Table 3). Indeed, this example only selects nine of the important factors in order to improve help-seeking attitudes as it is not yet clear which factors have a stronger influence on help-seeking than others. This example has been constructed through the use of the COM-B model, BCW, and specific BCTs to finalise a potential intervention.

Once an intervention has been designed, the acceptability and feasibility of the intervention should be evaluated. In this context, the MRC’s framework highlights the importance of evaluating the acceptability, compliance, delivery of the intervention, recruitment, and retention [26]. Here, we emphasise the importance of measuring the recruitment and retention to mental health initiatives whilst also evaluating the acceptability of help-seeking interventions for male students.

When investigating the evaluation methods from previous help-seeking interventions, there is not a consistent measure of acceptability. Across the 12 help-seeking interventions outlined in the development stage of this paper, only the Mental Health Ad Effectiveness Scale (MHAES) and the Treatment Evaluation Inventory Short Form (TEI-SF) have been used in one study each [23,24]. Despite both demonstrating good psychometric properties [37,104], the MHAES was designed to measure the effectiveness of brochures advertising mental health services [23], whilst the TEI-SF evaluates parents’ acceptance of interventions for behaviour problem children [104]. Subsequently, these are not suitable when evaluating mental health help-seeking interventions for male students.

To evaluate acceptability, the Theoretical Framework of Acceptability Questionnaire (TFAQ) was identified [105]. The TFAQ is a theory-informed questionnaire containing eight items evaluating the acceptability of healthcare interventions [105]. The eight items of the TFAQ capture eight distinct domains that relate to acceptability. These domains include general acceptability, affective attitude, burden, ethicality, intervention coherence, opportunity costs, perceived effectiveness, and self-efficacy [106]. Moreover, the TFAQ can be used within all four stages of the MRC’s framework for developing complex interventions and provides a more comprehensive definition of the term “acceptability”, synthesised from 43 review articles, allowing for better operationalisation [106]. As the TFAQ provides a general framework, it is possible to tailor this measure towards help-seeking in male students (Appendix C).

Once an intervention has been developed in accordance with this framework, it is recommended that the outcome measures of help-seeking attitudes and help-seeking behaviours are measured by the ATSPPH-SF and AHSQ, respectively. The final measure used to evaluate the feasibility and acceptability of a newly developed intervention is to use the TFAQ and make adaptive changes where necessary. Furthermore, newly developed interventions should be reported in accordance with the Template for Intervention Description and Replication (TIDieR) checklist to aid with replication and the clarity of the final intervention [22,33].

## 4. Strengths and Limitations

Here, the current paper provides an overview of the factors to embed within an intervention to improve mental health help-seeking for male students. The strengths of this paper are that it rigorously follows the MRC’s framework for developing a complex intervention. This allows for a detailed description of future interventions, enabling better replication, evidence synthesis, and wider implementation for researchers and health care professionals working with male students [26]. Another strength is that this framework makes use of other tools to improve the systematic nature of the recommendations provided. Here, the use of the COM-B model of behaviour change, BCW, BCTTv1 and APEASE criteria has been discussed when designing gender-sensitive interventions for male students with the ultimate goal to enhance their effectiveness and replicability once published [35,100,103]. Similarly, the use of the GUIDED checklist is provided to further enhance the description of this framework and allow readers to understand key aspects when developing mental health interventions for male students [25].

Despite these strengths, this paper is not without limitations. Although the current paper addresses mental health help-seeking for male-students specifically, some of the rationale underpinning key features are drawn from the adult male literature to provide a more comprehensive synthesis. Subsequently, the recommendations may not directly transfer to male-students. Indeed, younger adults are significantly less likely to seek help and hold more negative help-seeking attitudes [107,108], whilst students are also faced with barriers that may differ from non-students and older adult males. In an attempt to provide a comprehensive overview, the current paper is unable to provide more specific recommendations for sub-groups of male students. For instance, sexual minority male students or male students from ethnic minority backgrounds face different barriers and it is likely that they will need more tailored interventions to accommodate their needs and encourage help-seeking [109,110,111,112,113,114]. Lastly, this framework is yet to be implemented when designing future male-student help-seeking interventions. Although this paper synthesises evidence-based work specifically for men and male students, it is unclear as to how transferable and applicable this will be to real-world scenarios. Indeed, it would be valuable to see how effective/ineffective this framework is for others developing mental health interventions for male students.

## 5. Conclusions

Previous work has consistently identified that the onset of mental health difficulties, such as anxiety and depression often coincide with when students begin or start further education at university [2,3]. These mental health difficulties can be made worse from the pressures and expectations at university, contributing to a greater risk of suicide and protracted educational outcomes [4,5]. Typically, students and young people, irrespective of gender, are reluctant to seek help for mental health difficulties due to a range of barriers [11,15,16]. However, male students remain more reluctant to seek help for mental health due to additional barriers, such as traditional stereotypes of masculinity [12,13,17,18]. Due to male students being less likely to use mental health services and being at a higher risk of suicide than female students, universities are faced with an increased pressure to develop and implement effective initiatives for male students [21]. Nonetheless, such initiatives that have been developed often fail to be grounded in evidence-based practice or tailored to the needs of male students. Where such approaches have been implemented, the development process is not outlined. This creates significant difficulty for other healthcare or education providers to replicate, develop, or refine effective mental health initiatives that are tailored towards male students.

The current paper therefore provides an in-depth framework on how to develop and design mental health interventions for male students in accordance with the MRC’s framework for developing a complex intervention [26]. Indeed, this paper presents a series of recommendations that are grounded in evidence-based practice. Previous gender-sensitive help-seeking interventions for men and male students and their active ingredients (i.e., BCT’s) that are likely to elicit positive help-seeking attitudes or behaviours are first examined. Next, the identification of theory that is specific to male student’s help-seeking behaviour is outlined through the use of Gask’s access to care model [46]. By using previous published interventions and pre-existing theory further supplemented by qualitative findings from focus groups, 17 key factors that influence male students help-seeking for psychological support have been identified. These 17 factors allow for the operationalisation of key techniques that can be used to target help-seeking in male students. Through the use of the COM-B model of behaviour change, BCW, and BCTTv1, we have developed a framework for developing gender-sensitive interventions for male students that are likely to be effective and grounded in evidence-based practice. This paper also presents an example of an intervention that can be developed through the use of this framework to help inform future healthcare and education providers seeking to produce mental health interventions for male-students. It is hoped that this framework can be used to help reduce the gender disparity in those seeking mental health help can be reduced amongst a student population.

## Figures and Tables

**Table 1:** Summary of 17 factors influencing male students help-seeking for psychological support from various sources.

Factors Influencing Help-Seeking	Factors Targeted in Previous Interventions: Systematic Reviews (MRC 1.1)	Theory Relating to Men’s Help-Seeking: Access to Care Model (MRC 1.2)	Modelling Process and Outcomes: Focus Groups (MRC 1.3)
Help-seeking is not masculine	X	X	X
Public-stigma of help-seeking	X	X	X
Self-stigma of help-seeking	X	X	X
Difficulty identifying mental health symptoms	X	X	X
Unsure of treatment structure	X	X	X
Unfamiliarity with mental health services	X	X	X
Social support, support groups and occupational support	X	X	X
Current relationship with service provider (e.g., trust)		X	X
Symptom severity (i.e., delay until symptoms are unmanageable)		X	X
Preference for proactive therapies	X	X	
Availability of services (e.g., extended opening hours, during exams and freshers)		X	X
Ability to expressing emotions/emotional competence		X	
Structure of the intervention (i.e., formality and duration)			X
Past experience of help-seeking and current help-seeking attitudes		X	
Fear and embarrassment of using mental health services (treatment stigma)		X	
Treatment is too time consuming			X
Clinician difficulty in detecting male symptoms		X	
Clinician biases towards men with mental health difficulties		X	

**Table 2:** Mapping of help-seeking factors to a COM-B system of behaviour.

Capability: The Individual’s Capacity to Engage in the Behaviour	Opportunity: All Factors Lying Outside the Individual That Make Performance of the Behaviour Possible or Prompt it	Motivation: All Brain Process That Energise the Direct Behaviour
**Psychological**	**Physical**	**Reflective**
Difficulty identifying mental health symptoms	Availability of services	Help-seeking is not masculine
Ability to express emotions/emotional competence	Structure of the intervention	Self-stigma of help-seeking
Unsure of treatment structure	Preference for proactive therapies (availability)	Past experience of help-seeking
Unfamiliarity with mental health services	Treatment is too time consuming	Current help-seeking attitudes
Symptom severity (increases awareness)		Treatment stigma
		Symptom severity (evaluation of symptoms)
		Treatment is too time consuming (perception)
		Preference for proactive therapies (evaluation)
**Physical**	**Social**	**Automatic**
	Public stigma of help-seeking	
	Social support	
	Relationship with service provider	
	Clinician difficulty in detecting symptoms	
	Clinician biases	

**Table 3:** Example intervention for male students to improve mental health help-seeking, including Behavioural Change Techniques (BCTs).

Factor	COM-B Domain	Intervention Function	BCTs	Intervention Component
Difficulty identifying mental health symptoms	Psychological Capability	Education	2.2. Feedback on behaviour 5.1. Information about health consequences 5.3. Information about social and environmental consequences 5.6. Information about emotional consequences	Incorporate educational content that provides information about common mental health symptoms, their presentation, consequences of not seeking help, and use screening tools to assist students with self-identifying any current symptoms. This educational content can be delivered through a range of methods such as face-to-face classes, presentations, videos or educational leaflets.
Unsure of treatment structure	Psychological Capability	Education	5.1. Information about health consequences 5.6. Information about emotional consequences	Provide information about how service referrals and assessments operate. This may include information pertaining to waiting lists and where the referral takes place. Outline the treatment structure such as the number of sessions, how long appointments last for, and the types of confidentiality across services. Information can be delivered through a range of methods including face-to-face classes, presentations, videos or educational leaflets.
Unfamiliar with mental health services	Psychological Capability	Education	3.1. Social support (unspecified) 3.2. Social support (practical)	Explain and sign-post different mental health services and support options. This includes the names of different services, the types of support they would receive and the geographical location of such support. Information can be delivered through a range of methods including face-to-face classes, presentations, videos or educational leaflets.
Social support	Social Opportunity	Environmental Restructuring	3.1. Social support (unspecified)	Advise students to talk to friends and family about their mental health or provide environments that are conducive to forming social relationships. Advice can be delivered though presentations, posters, videos or educational leaflets.
Preference for proactive therapies	Psychological Capability or Reflective Motivation	Environmental Restructuring	1.2. Problem solving 1.4. Action planning 11.2. Reduce negative emotions	Incorporated self-management strategies such as relaxation, time management, problem solving, and action planning to resolve mental health difficulties. Such strategies can be delivered in face-to-face class sessions or group settings. Referral to (online) self-help materials or video resources may also be suitable.
Help-seeking is not masculine	Reflective motivation	Modelling	6.2. Social comparison 9.1. Credible source 13.2. Framing/Re-framing	Use group settings to discuss how mental health can still be masculine (e.g., a sign of strength). Draw attention to male celebrities and male role models who have sought help and are successful. Alternatively, use posters, videos or leaflets to promote help-seeking as a masculine trait.
Self-stigma of seeking help	Reflective Motivation	Modelling	6.2. Social comparison 13.2. Framing/Re-framing	Reframe help-seeking to be positive and provide examples of others with mental health difficulties and how seeking help improved their well-being. Reframing can be achieved through group discussions, presentations, leaflets, posters or videos.
Treatment-stigma	Reflective Motivation	Persuasion	5.1. Information about health consequences 5.6. Information about emotional consequences	Outline the benefits of treatment and what can be achieved if engaged with. Draw particular attention to one’s well-being, reduction of symptoms, and increased functioning. Information can be delivered through a range of methods including face-to-face classes, presentations, videos or educational leaflets.
Structure of the intervention	Physical Opportunity	Environmental Restructuring	NA	Create a male-only space for students to drop-in to as opposed to a formal intervention. Here, this drop-in space could be more attractive to male students and make the intervention less time consuming. Physical spaces that have a central theme (e.g., sports or arts and crafts) are likely to appeal to male students. However, online male spaces (e.g., gaming) may provide a similar opportunity.

## Appendix A

Guidance for Reporting Intervention Development Studies in Health Research (GUIDED) checklist.

**Item**	**Description**	**Manuscript**
1. Report the context for which the intervention was developed.	Understanding the context in which an intervention was developed informs readers about the suitability and transferability of the intervention to the context in which they are considering evaluating, adapting or using the intervention.	“It is essential for the intervention development process to be reported as this can enhance our theoretical and practical understanding about developing mental health interventions for male students [25]”. In response to this, the current paper seeks to develop the first framework for developing and designing mental health interventions for male students that is grounded in evidence-based practice.
2. Report the purpose of the intervention development process.	Clearly describing the purpose of the intervention specifies what it sets out to achieve. The purpose may be informed by research priorities, for example those identified in systematic reviews, evidence gaps set out in practice guidance, such as the NICE, or specific prioritisation exercises.	“The MRC framework for developing and evaluating complex interventions will be used with the Behaviour Change Wheel to develop a framework for new interventions that address help-seeking in male students. It is anticipated that this framework will create a starting point for future interventions which can be refined as the current evidence base is enriched. Furthermore, specific detail in accordance to the Guidance for Reporting Intervention Development Studies in Health Research (GUIDED) checklist has been included (Appendix A) to further enrich the quality of evidence that is reported within the current paper [25].”
3. Report the target population for the intervention development process.	The target population is the population that will potentially benefit from the intervention–this may include patients, clinicians and/or members of the public. If the target population is clearly described, then readers will be able to understand the relevance of the intervention to their own research or practice. Health inequalities, gender and ethnicity are features of the target population that may be relevant to the intervention development process.	“In response to this, the current paper seeks to develop the first framework for developing and designing mental health interventions for male students that is grounded in evidence-based practice.”
4. Report how any published intervention development approach contributed to the development process.	Many formal intervention development approaches exist and are used to guide the intervention development process. Where a formal intervention development approach is used, it is helpful to describe the process that was follows, including any deviations.	“This paper will discuss the development of an intervention using a published approach grounded in theory and evidence base by combining published research evidence and existing theories [27]. The MRC framework for developing and evaluating complex interventions will be used with the Behaviour Change Wheel to develop a framework for new interventions that address help-seeking in male students.”
5. Report how evidence from different sources informed the intervention development process.	Intervention development is often based on published evidence and/or primary data that have been collected to inform the intervention development process. It is useful to describe and reference all forms of evidence and data that have informed the development of the intervention because evidence bases can change rapidly, and to explain the manner in which the evidence and/or data were used.	“This paper will discuss the development of an intervention using a published approach grounded in theory and evidence base by combining published research evidence and existing theories [27].”This paper also incorporates published systematic reviews and qualitative findings from focus groups into the development of the intervention throughout the paper.
6. Report how/if existing published theory informed the intervention development process.	Reporting whether and how theory informed the development process aids the reader’s understanding of the theoretical rationale that underpins the intervention. This can relate to either existing published theory or programme theory.	This is utilised throughout the paper. The paper draws upon published evidence from systematic reviews, qualitative findings from focus groups and theory informed research structured within the ‘access to care model’.
7. Report any use of components from an existing intervention in the current intervention development process.	Some interventions are developed with components that have been adopted from existing interventions. Clearly identifying components that have been adopted or adapted and acknowledging their original source helps the reader to understand and distinguish between the novel and adopted components of the new intervention.	“Sagar-Ouriaghli and colleagues [22] identified 18 BCTs (e.g., credible source, feedback on behaviour and problem solving), which were in turn synthesised into broader, more clinically relevant, psychological processes that are likely to contribute to changes in help-seeking for men of different age groups (Appendix B). These seven key processes include: the use of role models (e.g., celebrities and other men) to convey information, psycho-educational materials to improve mental health knowledge, assisting men to recognise and manage their symptoms, adopting active problem solving and/or solution focused tasks, motivating behaviour change, sign-posting mental health services and finally, including content to build on positive masculine traits (e.g., responsibility and strength).”
8. Report any guiding principles, people or factors that were priorities when making decisions during the intervention development process.	Reporting any guiding principles that governed the development of the intervention will help the reader to understand the authors’ reasoning behind the decisions that were made. Guiding principles specify the core objectives and features of the desired intervention.	“Lastly, not all intervention functions should be implemented, and should be chosen based on their affordability, practicability, effectiveness/cost-effectiveness, acceptability, side-effects/safety and equity–otherwise known as the APEASE criteria [103]. … Once all potential BCT’s have been identified, the APEASE criteria is used once more to determine which specific techniques or tools are most appropriate. Additionally, BCT’s that have been frequently used before in similar interventions may also aid in this decision [22,103].”
9. Report how stakeholders contributed to the intervention development process.	Potential stakeholders can include patient and community representatives, local and national policy makers, healthcare providers, and those paying for or commissioning healthcare. Each of these groups may influence the intervention development process in different ways.	The intervention development outlines how the theory informed components are also based on focus group findings from the potential service users of a male-student intervention for mental health help-seeking.
10. Report how the intervention changed in content and format from the start of the intervention development process.	Due to the iterative nature of intervention development, the intervention that is defined in the end of the development process can often be quite different from the one that was initially planned. Describing these changes and their rationale enhances understanding and enables understanding other intervention developers to learn from this experience.	This is not applicable to the current paper. Here, the current paper outlines key factors that are likely to be important that can be used as a template/framework for future interventions. In the instance that interventions are developed following this framework, any changes to the intervention/deviation from the proposed framework should be reported here.
11. Report any changes to interventions required or likely to be required for subgroups.	Specifying any changes that the intervention development team perceive are required for the intervention to be delivered or tailored to specific subgroups enables readers to understand the applicability of the intervention to their target population or context.	This is not applicable to the current paper. Here, the current paper seeks to provide a broad overview of the factors which are likely to be effective when designing mental health interventions for male students. Therefore, broad intervention strategies are outlined. If a more specific sub-group of male students is the focus of a newly proposed intervention this should be stated in future work.
12. Report important uncertainties at the end of the intervention development process.	Intervention development is frequently an iterative process. The conclusion of the initial phase of intervention development does not necessarily mean that all uncertainties have been addressed. It is helpful to list remaining uncertainties, such as the intervention intensity, mode of delivery, materials, procedures or type of location that the intervention is most suitable for. This can guide other researchers to potential future areas of research and practitioners about uncertainties relevant to their healthcare context.	“Subsequently, the recommendations may not directly transfer to male-students. Indeed, younger adults are significantly less likely to seek help and hold more negative help-seeking attitudes [107,108], whilst students are also faced with barriers which may differ from non-students and older adult males. In an attempt to provide a comprehensive overview, the current paper is unable to provide more specific recommendations for sub-groups of male students. For instance, sexual minority male students or male students from ethnic minority backgrounds face different barriers and it is likely that they will need more tailored interventions to accommodate their needs and encourage help-seeking [109,110,111,112]. Lastly, this framework is yet to be implemented when designing future male-student help-seeking interventions. Although this paper synthesises evidence-based work specifically for men and male students, it is unclear as to how transferable and applicable this will be to real world scenarios. Indeed, it would be valuable to see how effective/ineffective this framework is for others developing mental health interventions for male students.”
13. Follow TIDieR guidance when describing the developed intervention.	Interventions have been poorly reported for a number of years. In response to this, internationally recognised guidance has been published to support the high-quality reporting of healthcare interventions and public health interventions. This guidance should therefore be followed when describing a developed intervention.	This is not applicable to the current paper as it does not report a specific intervention, nor does it pilot the described intervention. However, the current paper recommends the use of TIDieR when interventions are designed in accordance to this framework. Furthermore, newly developed interventions should be reported in accordance to the Template for Intervention Description and Replication (TIDieR) checklist to aid with replication and clarity of the final intervention [22,33].”
14. Report the intervention development process in an open access format.	Unless reports of intervention development are available, people considering using an intervention cannot understand the process that undertaken and make a judgement about its appropriateness to their context. It also limits cumulative learning about intervention development mythology and observed consequences at later evaluation, translation and implementation stages. Reporting intervention development in an open access publishing format increases the accessibility and visibility of intervention development research and makes it more likely to be read and used.	The current paper was submitted and published in open access format in the *International Journal of Environmental Research and Public Health* (*IJERPH*).

## Appendix B

Summary of BCTs and processes identified in male help-seeking interventions. Adapted from Sagar-Ouriaghli et al. (2019) [22].

## Appendix C

Theoretical Framework of Acceptability Questionnaire (TFAQ)

**Instructions:** Read each question carefully and indicate your response using the scale below (1-2-3-4-5).

No.	Item				
1.	How acceptable was the workshop?				
	Completely Unacceptable	Unacceptable	No Opinion	Acceptable	Completely Acceptable
	1	2	3	4	5
2.	Did you like or dislike the workshop?				
	Strongly Dislike	Dislike	No Opinion	Like	Strongly Like
	1	2	3	4	5
3.	How much effort did it take you to engage with the workshop?				
	No effort at all	A little effort	No Opinion	A lot of effort	Huge effort
	1	2	3	4	5
4.	The workshop fits with my beliefs about mental health and seeking mental health support				
	Strongly Disagree	Disagree	No Opinion	Agree	Strongly Agree
	1	2	3	4	5
5.	It is clear to me how engaging in this workshop would help me manage my mental health				
	Strongly Disagree	Disagree	No Opinion	Agree	Strongly Agree
	1	2	3	4	5
	Please tell us more about your views				
	

6.	This workshop interfered with my other priorities				
	Strongly Disagree	Disagree	No Opinion	Agree	Strongly Agree
	1	2	3	4	5
7a.	The workshop has improved my attitudes towards seeking professional help for my mental health				
	Strongly Disagree	Disagree	No Opinion	Agree	Strongly Agree
	1	2	3	4	5
7b.	The workshop has improved my overall mental health/well-being				
	Strongly Disagree	Disagree	No Opinion	Agree	Strongly Agree
	1	2	3	4	5
8.	How confident would you feel about engaging with this workshop again?				
	Very Unconfident	Unconfident	No Opinion	Confident	Very Confident
	1	2	3	4	5
